# Conditioned Flavor Preference and the US Postexposure Effect in the House Musk Shrew (*Suncus Murinus*)

**DOI:** 10.3389/fpsyg.2012.00242

**Published:** 2012-07-16

**Authors:** Kosuke Sawa, Kiyoshi Ishii

**Affiliations:** ^1^Department of Psychology, Senshu UniversityKawasaki, Kanagawa, Japan; ^2^Department of Social and Human Environment, Nagoya UniversityNagoya, Aichi, Japan

**Keywords:** house musk shrews, associative learning, conditioned flavor preference, US postexposure effect, habituation

## Abstract

The house musk shrew (*Suncus murinus*) is the only species of mammalian insectivore that can be domesticated and used as a laboratory animal, and is an interesting subject in terms of evolutionary and comparative aspects. The present study on the learning faculties of shrews examines the possibility of acquiring a conditioned flavor preference and the effects of US postexposure. Subjects were allowed to a drink sucrose solution with flavor A and tap water with flavor B during training. Two extinction tests were administered after every four conditioning trials, and a significant preference for flavor A was observed. After each test, the animals were divided into two groups. Subjects in Group US were presented with a sucrose solution without flavor, while those in Group Water were given tap water. After these trials, all subjects received choice tests where they were presented with water containing the two flavors. The preference ratio was lower in Group US than in Group Water, suggesting a postexposure effect. The findings were discussed in terms of habituation to the US.

## Introduction

The house musk shrew (*Suncus murinus*) is the only species of mammalian insectivore that has been domesticated successfully and used as a laboratory animal. As insectivores are thought to be common ancestors of present mammals, it is important to keep evolutionary and comparative aspects in mind when investigating the cognitive properties of shrews (for a review, see Tsuji et al., 1999). Despite the implications that the findings of studies on shrews may have on mammalian cognitive properties, shrews have been given little attention in psychological research and in learning studies in particular. Less than 50 studies with the key term “*Suncus murinus*” as query were found in PsychINFO, whereas over 40,000 studies were found with the term “rat” at June 16, 2012.

House musk shrews can be found in relatively low-latitude areas, from eastern Africa to southwestern Japan. Since they prey primarily on insects and worms in the natural environment, the problems in food selection they encounter seem to be simple compared to those faced by omnivores, including rats and humans, who have to consume a variety of foods to survive and thus face problems in choosing appropriate foods and avoiding poisonous ones. The conditioned taste aversion (Garcia and Koelling, 1966) is an important adaptation evolved to solve the problem of avoiding poisonous foods, and has been examined in various situations and species (Reilly and Schachtman, 2009). The basic procedure of a conditioned taste aversion experiment consists of subjects receiving the pairing of the taste stimulus and a poisonous treatment such as an injection of LiCl. After these training trials, subjects acquire an aversive response to the taste stimulus. Since the avoidance of poisonous substances is vital for survival in the natural environment for insectivores, conditioned taste aversion has been studied and confirmed in house musk shrews (e.g., Smith et al., 2001).

However, omnivores face another problem regarding food selection. Because they have to eat a wide variety of foods, they need to choose foods high in nutrition, which leads to an innate preference for sweet foods (typically signaling high caloric content) in many species. Conditioned flavor preference (Capaldi, 1991; Sclafani, 1991 for review), another adaptation for food selection, helps organisms to learn associations between flavors of food and nutrition level. Several studies have demonstrated that many organisms show a preference for flavors that are paired with sweet or nutritional substances like sucrose, saccharin, ethanol, and starch (e.g., Holman, 1975; Fedorchak and Bolles, 1987; Ramirez, 1994). Conditioned flavor preference, together with conditioned taste aversion, fall under a type of Pavlovian conditioning (Pavlov, 1927), where an association between a flavored solution as the conditioned stimulus (CS) and a sweet/nutritional or poisonous substance as the unconditioned stimulus (US) is formed. The standard procedure for conditioned flavor preference is as follows: subjects undergo conditioning trials where they drink both a sucrose solution with artificial flavor A and tap water with flavor B (A+/B−). They then undergo an extinction test where they choose between the two flavors, with both added to tap water (A−/B−). If an association of flavor A with sucrose is established during conditioning, subjects should show a preference for flavor A over flavor B in the extinction test.

The only study, as far as we know, that deals with conditioned taste preference in shrews was conducted by Parker et al. (2002). They found that using rewarding drugs like morphine as the US produced a conditioned taste preference to the CS in shrews, whereas rats that underwent the same procedure developed a rather aversive response to the CS (see also, e.g., Hunt and Amit, 1987). Although this result implies that the acquisition of conditioned taste preference is possible in shrews, there has been no research conducted where a sweet taste functions as the US. Since shrews show a considerable preference for sweet tastes, similar to rats (Iwasaki and Sato, 1982), a sucrose solution at a particular concentration may work effectively as the US, even though shrews cannot digest sucrose (Yokota et al., 1992). Therefore, one aim of the present study is to examine the possibility of acquiring a conditioned flavor preference with a sweet taste as the US in house musk shrews by using the standard procedure with a within-subject design in order to extend the existing literature on this behavior in an overlooked species.

Another aim of this study is to investigate US habituation through training, as well as the US postexposure effect on conditioned flavor preference in shrews. During food selection, organisms tend to exhibit neophobic responses, where they eat relatively small amounts of novel foods. This behavioral tendency serves to protect the organism by preventing the consumption of large amounts of a potentially poisonous substance. The amount of food intake increases with each experience of consuming the food without any adverse effects, and this process has been interpreted as habituation (Kaye et al., 1988). For omnivores, which have a bigger food selection, neophobic responses and habituation processes are adaptive behaviors designed to learn which food is safe for consumption. On the other hand, insectivores have a comparatively small selection of food, resulting in smaller adaptive values of neophobic responses and habituation processes in food selection. If this conjecture is true, neophobic responses in shrews should be weaker and habituation processes slower compared to those of omnivores such as rats. In the present study, we will also investigate the effect of US habituation on conditioned preference after training, by presenting the US without the CS (Rescorla, 1973). Exposing the subjects to the US after they have acquired considerable CR often causes a significant decline in the CR. This phenomenon is known as the US postexposure effect (see Delamater and LoLordo, 1991 for a review). Rescorla (1973) suggested that the habituation of the representation of the US during US postexposure trials brought about the attenuation of CR. US postexposure has been found to attenuate the conditioned response (CR) in conditioned flavor preference as well (e.g., Boakes et al., 2007; Kawai and Nakajima, 1997). By administering US postexposure trials, the present study investigates habituation effects in shrews from multiple points of view.

## Materials and Methods

### Subjects and apparatus

The subjects were seven male and seven female naïve house musk shrews, mean weighing 36.5 g at the beginning of experimental treatment. Each subject was housed in an individual cage (25 cm × 19 cm × 13 cm) and maintained on a 12:12-h light-dark cycle with an *ad lib* food and water schedule unless otherwise noted. All the experimental treatments were carried out in the individual cages, and fluid (tap water or sucrose solution with artificial flavors) was provided via a plastic bottle with a metal spout. This research was conducted following the relevant ethics guidelines for research with animals, and was approved by Nagoya University’s IACUC.

### Procedure

A within-subject design as summarized in Table 1 was adopted for the experiment. On each day of the experimental period, food and water were withheld at 11:00, and the experimental trial was carried out at 23:00. After the experimental trial, subjects were allowed to access food and water freely until 11:00 of the next day. During the initial 3 days (days 1–3), all subjects were allowed to drink tap water for 30 min in their home cages. For the next 4 days (days 4–7), they were exposed to flavored 0.3 M sucrose solution and flavored tap water simultaneously for 30 min. Two bottles were located 16 cm apart with each other diagonal across the cage. The main reason why we adopt two-bottle method for conditioning was to enhance contrast between solutions and strengthen conditioning. For one half of the subjects, an almond flavor (GABAN-Asaoka, Japan) was paired with the sucrose solution and a lemon flavor (GABAN-Asaoka, Japan) was paired with tap water; the pairings were reversed for the other half of the subjects. Flavoring was accomplished by adding 0.5 mL of the artificial flavor to 50 mL of sucrose solution or tap water. On day 8, a preference test was carried out. The procedure was identical to that used in conditioning, except that both flavors were presented with tap water (A−/B−). These treatments, consisting of a 5-day cycle (4 days for conditioning and 1 day for the preference test), were administered twice. Thus, the procedure during days 9–12 (conditioning 2) was identical to that of days 4–7 (conditioning 1), and the procedure on day 13 (test 2) was the same as that of day 8 (test 1).

**Table 1:** **Design table of conditioned flavor preference during training and testing**.

Training	First cycle		Second cycle	
	Conditioning	Test 1	Conditioning	Test 2
Days 1–3	Days 4–7	Day 8	Days 9–12	Day 13
Water	A+/B−	A−/B−	A+/B−	A−/B−

*A and B, almond or lemon flavor counterbalanced; +, 0.3 M sucrose; −, tap water*.

After the second test of conditioned flavor preference, the subjects were divided into two equal-sized groups labeled Group US and Group Water. The two groups were matched in terms of preference ratio (A− consumed/A− + B− consumed). On days 14 and 15, the subjects in Group US were allowed to consume 0.3 M sucrose for 30 min as postexposure to the US (see Table 2). The subjects in Group Water were allowed to drink tap water in the same manner. After these sessions, testing was conducted on day 16 in the A−/B− fashion as described above.

**Table 2:** **Design table of US postexposure treatment and testing**.

Groups	Days 14 and 15	Day 16
US	Sucrose	A−/B−
Water	Water	A−/B−

*A and B, almond or lemon flavor counterbalanced; −, tap water*.

Throughout the experiments, the amount of fluid intake was measured by weighing each bottle to the nearest 0.01 g by using an electronic balance. The position of bottles was balanced over training and testing across subjects throughout the experiment.

## Results

**Figure 1:**
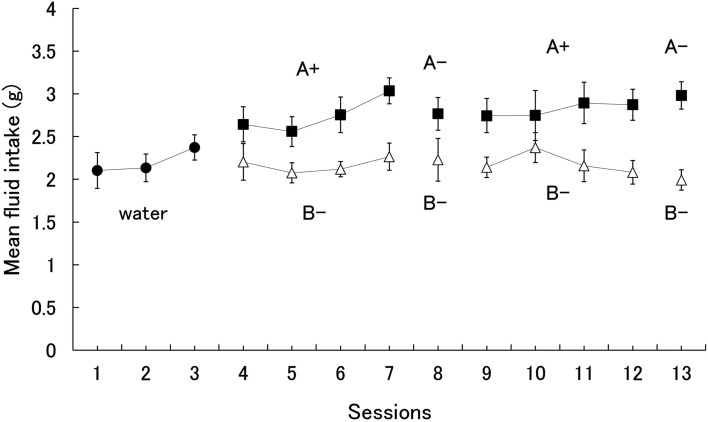
**Mean intake of each stimulus during acquisition and probe test of conditioned flavor preference**. Error bars indicate standard errors.

Since the subjects did not show a significant preference for one flavor over the other when both were paired with sucrose, the results show only the mean fluid intake for the 14 subjects. The alpha level was set at 0.05 unless otherwise noted. Figure 1 shows the mean amount of fluid intake during all experimental sessions. A one-way analysis of variance (ANOVA) on water consumption for days 1–3 yielded no significant effect, *F*(2,26) = 1.71. During the first conditioning phase (days 4–7), however, subjects were found to consume the A+ solution more than B−. A two (stimuli) × four (session) ANOVA yielded significant main effects of session, *F*(3,39) = 4.55, and stimuli, *F*(1,13) = 27.56. Subsequent Fisher’s LSD tests revealed that the amount of intake on Day 7 was greater than on each of the other days.

Our main interest was the shrews’ preference for A− during the extinction test on day 8. Subjects drank more of the A− than B− solution (Figure 1). However, statistical analysis did not yield a significant difference, *F*(1,13) = 1.94.

A two (stimuli) × four (session) ANOVA conducted for the second conditioning phase (days 9–12) yielded a significant main effect of stimuli, *F*(1,13) = 7.20, similar to the first phase. During the extinction test on day 13, subjects drank more A− solution than B−. A one-way ANOVA yielded a significant effect, *F*(1,13) = 21.86, indicating that the shrews took approximately eight sessions to acquire a preference for the flavor paired with the sucrose solution.

The greater consumption of the solution tinged with flavor A during the conditioning phase could be a result of its pairing with sucrose, facilitating familiarity of flavor A, and thus resulting in its apparent preference over B. If individual differences concerning the consumption of A are directly affected by the degree of preference in the test phase, there should be a significant correlation of an individual’s consumption of flavor A between the conditioning phase and the test phase. However, correlation analysis between the mean intake of A+ during the conditioning phases (days 4–7, 9–12) and A− during the test trial (day 13) revealed no significant correlation (*r* = −0.03), suggesting that a familiarity effect was not responsible for the result.

**Figure 2:**
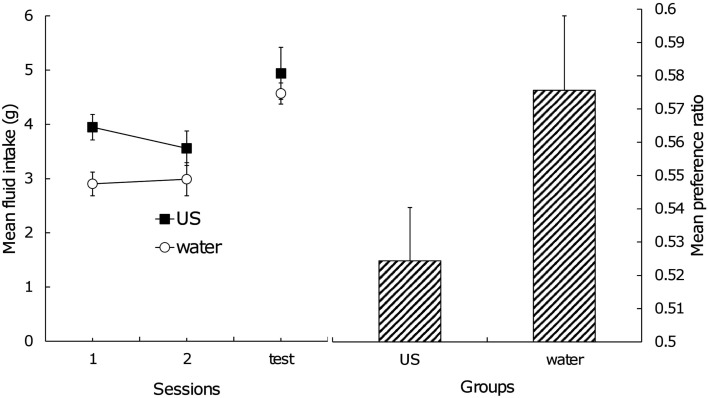
**Left: mean total intake of stimuli across US postexposure phase and test trial, Right: mean preference ratio for flavor A in test trial**. Error bars indicate standard errors.

The left side of Figure 2 shows the mean fluid intake during the postexposure phase and test trial. Clearly, Group US had a greater amount of fluid intake than Group Water during the postexposure phase because of an innate preference for sucrose. A two (group) × two (session) factorial ANOVA yielded a significant main effect of group, *F*(1,12) = 7.81, confirming the above conjecture. In the test trial, the two groups displayed almost the same amount of intake; a one-way ANOVA did not obtain a significant difference between them, *F*(1,12) < 1. The direct index of the effect of US postexposure should be the amount of fluid consumption of each stimulus during testing. Mean consumption of flavor A in Group US was 2.60 g (standard error = 0.27) and flavor B was 2.34 g (SE = 0.23), and in Group Water, 2.63 g (SE = 0.15) and 1.94 g (SE = 0.14) for flavor A and B, respectively. A two (group) × two (fluid) factorial ANOVA yielded no significant main effect of group and interaction, presumably because of large individual difference. Significant main effect of fluid was found, *F*(1, 12) = 13.30, suggesting that subjects consumed flavor A more than flavor B and that conditioned flavor preference itself was still intact. In order to rule out the effect of individual difference of motivation on the amount of fluid intake and to focus on how extent the subjects prefer the CS+ (i.e., flavor A) relative to the CS− (i.e., flavor B), the preference ratio was calculated. The right side of Figure 2 indicates the mean preference ratio for flavor A in the test trial. A one-way ANOVA yielded a significant effect, *F*(1,12) = 3.41, with a one-tailed test, suggesting that US postexposure attenuated the CR although the effect was weak.

## Discussion

The present results suggest that shrews can acquire conditioned flavor preference with a simple but standard method. Our results are consistent with those obtained in previous studies using rats as subjects. However, a relatively higher number of trials (eight) were required to establish conditioned flavor preference in shrews; in previous research conducted by Kawai and Nakajima (1997) and Sawa et al. (1998) using a similar method, rats showed significant flavor preference after four trials. In addition, shrews showed a relatively weak preference compared to rats; Kawai and Nakajima (1997) reported a nine preference ratio in rats.

Rats generally display a neophobic response to novel tastes, even toward palatable tastes such as sweetness, and this response decreases as stimulus presentation is repeated (i.e., habituation). On the other hand, the shrews in our study did not seem to consume the novel sucrose solution in increasing amounts during the conditioning phase (Figure 1), although they did increase their intake on day 7, a finding suggesting the loss of the neophobic response. While it would be reasonable to assume that the habituation process was responsible for this increase in consumption, the amount of intake on day 7 was equivalent to that in the second cycle (days 9–12). Indeed, this pattern of results imply the lack of a neophobic response in the house musk shrew since rats in similar studies (e.g., Kawai and Nakajima, 1997) did not show such consistent levels of intake throughout their sessions. Parker et al. (2002) also reported no evidence of shrews exhibiting neophobic reactions to 0.3 M sucrose solutions, which were used in our study as the US. Ishii et al. (2000) reported that thirsty shrews continued showing a phobic response to a 0.5% vinegar solution that did not habituate even after 14 sessions. All of these studies suggest that the amount of novel fluid consumed does not increase with increasing exposure in shrews, indicating that shrews do not habituate to novel taste stimuli.

Undoubtedly, shrews can acquire a preference for a flavor paired with sucrose. However, as described above, the strength of the preference was less than that shown in rats. For rats, saccharin, which holds no nutritional value, could serve as the US in conditioned flavor preference (Holman, 1975). However, a palatable taste without any nutritional value is a relatively weaker reinforcer than that associated with a nutritional value (also see Capaldi, 1991). Considering that disaccharides like sucrose are indigestible by shrews, the present observation of a slow acquisition of conditioned flavor preference in shrews was a consequence of a flavor-taste association rather than a flavor-nutrition association. Therefore, our results regarding the US postexposure treatments, where the CR was attenuated after exposing the subjects to sucrose following conditioning, can also be interpreted as a consequence of habituation to the sweet taste.

Alternatively, our results could be interpreted using the associative learning theory. According to the comparator hypothesis (Miller and Matzel, 1988; Miller and Schachtman, 1985), the attenuation of the CR by exposing subjects to the US without the CS after conditioning is due to a strengthening of the association between the US and the training context. Randich and Rescorla (1981) compared the degree of attenuation of the CR in a group that received the US alone following conditioning with that in a group that received the US paired with another CS. If the context-US association plays an important role in the US postexposure effect, adding a novel CS during the US postexposure phase should result in overshadowing of the context by this new CS, leading to an interference with the attenuation of the CR. However, in their study, Randich and Rescorla (1981) found that these two groups showed the same degree of attenuation, suggesting that the US-context association is not critical in attenuating the CR. Furthermore, in our experiment, the subjects received all experimental treatments in their home cages, extinguishing the US-context association. This also implies that the US-context association does not contribute to the postexposure effect.

Another possible explanation of our results is satiation. Fedorchak and Bolles (1987) pointed out that food satiation treatment after flavor-nutrient learning reduces conditioned flavor preference. The level of hunger drive during the test trial may be a critical factor for the preference to emerge (Capaldi et al., 1994; see also Coldwell and Tordoff, 1993). If an exposure to sucrose caused satiation, subjects in Group US should decrease the amount of fluid intake in the test trial. However, as the left side of Figure 2 shows, the two groups did not differ from each other in this aspect. Thus, US habituation, rather than satiation, provides a more adequate explanation for our results.

Although the present study indicates that the house musk shrew, an insectivore, could acquire conditioned flavor preference and demonstrate habituation, these effects were generally small. As mentioned in the introduction, this may be due to an insectivore’s food selection strategy. Conditioned flavor preference is a process that helps organisms to select foods high in nutrition by forming an association between flavor and taste/nutrition, and habituation allows organisms to ignore stimuli that do not predict the occurrence of a biologically significant event. The house musk shrews, however, just have smaller variety of selecting food relative to the omnivores, implying the system of conditioned flavor preference and habituation to novel food seems to provide relatively small adaptive benefit for shrews in terms of food choice. On the other hand, previous research (Parker et al., 2002) obtaining conditioned taste preference in the house musk shrews used amphetamine and morphine as the USs, which have strong pharmacological impact on organism. It may be possible that shrews could form association between taste and pharmacological effect although nutritional effect, using in present research, has weak impact. It is difficult to affirm, however, that the same mechanisms underlie associative learning and habituation in both shrews and rats. Hence, in future, it would be beneficial to continue to compare the mechanisms that underlie these behaviors between these two species.

## Conflict of Interest Statement

The authors declare that the research was conducted in the absence of any commercial or financial relationships that could be construed as a potential conflict of interest.
